# Supplementary material for: Differences in arousal and valence on the Korean phoneme of artificial voice between Korean and Chinese women
Source: PLoS One. 2023 Apr 7;18(4):e0284045. doi: 10.1371/journal.pone.0284045 (PMC10081760; doi:10.1371/journal.pone.0284045)
Supplement: Attachment — Submitted filename: Response to Reviewers.docx [file pone.0284045.s002.docx]

**Editor’s requirement**

1. Please ensure that your manuscript meets PLOS ONE's style requirements, including those for file naming. The PLOS ONE style templates can be found at https://journals.plos.org/plosone/s/file?id=wjVg/PLOSOne_formatting_sample_main_body.pdf and <https://journals.plos.org/plosone/s/file?id=ba62/PLOSOne_formatting_sample_title_authors_affiliations.pdf>

A) This paper was written by referring to the writing method and form.

2. Please provide additional details regarding participant consent. In the ethics statement in the Methods and online submission information, please ensure that you have specified (1) whether consent was informed and (2) what type you obtained (for instance, written or verbal, and if verbal, how it was documented and witnessed). If your study included minors, state whether you obtained consent from parents or guardians. If the need for consent was waived by the ethics committee, please include this information. If you are reporting a retrospective study of medical records or archived samples, please ensure that you have discussed whether all data were fully anonymized before you accessed them and/or whether the IRB or ethics committee waived the requirement for informed consent. If patients provided informed written consent to have data from their medical records used in research, please include this information.

A) We mentioned about the participant consent in procedure part as following: All participants were able to start an online survey after agreeing to participate in the survey. In the first page of online survey form, they were informed about the research procedure, the possible risks, and the right to voluntarily stop participating in the experiment in writing.

3. Thank you for stating the following financial disclosure:“This work was supported by the Ministry of Education of the Republic of Korea and the National Research Foundation of Korea(NRF-2019S1A5C2A04082405)” Please state what role the funders took in the study.  If the funders had no role, please state: "The funders had no role in study design, data collection and analysis, decision to publish, or preparation of the manuscript." If this statement is not correct you must amend it as needed. Please include this amended Role of Funder statement in your cover letter; we will change the online submission form on your behalf.

A) We deleted funding statement in manuscript. And we mentioned this information in revised cover letter.

4. Thank you for stating the following in the Acknowledgments Section of your manuscript: “This work was supported by the Ministry of Education of the Republic of Korea and the National Research Foundation of Korea (NRF-2019S1A5C2A04082405)”. We note that you have provided additional information within the Acknowledgements Section that is not currently declared in your Funding Statement. Please note that funding information should not appear in the Acknowledgments section or other areas of your manuscript. We will only publish funding information present in the Funding Statement section of the online submission form. Please remove any funding-related text from the manuscript and let us know how you would like to update your Funding Statement. Currently, your Funding Statement reads as follows: “This research was supported by the Ministry of Education of the Republic of Korea and the National Research Foundation of Korea (NRF-2019S1A5C2A04082405)”. Please include your amended statements within your cover letter; we will change the online submission form on your behalf.

A) We deleted funding statement in manuscript. And we mentioned this information in revised cover letter.

5. We note that you have indicated that data from this study are available upon request. PLOS only allows data to be available upon request if there are legal or ethical restrictions on sharing data publicly. For more information on unacceptable data access restrictions, please see <http://journals.plos.org/plosone/s/data-availability#loc-unacceptable-data-access-restrictions>. In your revised cover letter, please address the following prompts: a) If there are ethical or legal restrictions on sharing a de-identified data set, please explain them in detail (e.g., data contain potentially sensitive information, data are owned by a third-party organization, etc.) and who has imposed them (e.g., an ethics committee). Please also provide contact information for a data access committee, ethics committee, or other institutional body to which data requests may be sent. b) If there are no restrictions, please upload the minimal anonymized data set necessary to replicate your study findings as either Supporting Information files or to a stable, public repository and provide us with the relevant URLs, DOIs, or accession numbers. For a list of acceptable repositories, please see <http://journals.plos.org/plosone/s/data-availability#loc-recommended-repositories>. We will update your Data Availability statement on your behalf to reflect the information you provide.
A) There are no restrictions. And we mentioned it in revised cover letter.
6. PLOS requires an ORCID iD for the corresponding author in Editorial Manager on papers submitted after December 6th, 2016. Please ensure that you have an ORCID iD and that it is validated in Editorial Manager. To do this, go to ‘Update my Information’ (in the upper left-hand corner of the main menu), and click on the Fetch/Validate link next to the ORCID field. This will take you to the ORCID site and allow you to create a new iD or authenticate a pre-existing iD in Editorial Manager. Please see the following video for instructions on linking an ORCID iD to your Editorial Manager account: [https://www.youtube.com/watch?v=_xcclfuvtxQ](https://www.youtube.com/watch%5C?v=_xcclfuvtxQ)

A) The corresponding author already has an ORCID iD.

7. We note that you have referenced (ie. Bewick et al. [5]) which has currently not yet been accepted for publication. Please remove this from your References and amend this to state in the body of your manuscript: (ie “Bewick et al. [Unpublished]”) as detailed online in our guide for authors <http://journals.plos.org/plosone/s/submission-guidelines#loc-reference-style>
A) We didn’t have referenced Bewick et al., study.

**Response to the Reviewer #1**

1. In the abstract and other part of your paper, I suggest to use consonant, instead of articulation or articulation methods to avoid confusion. In the field of Phonetics Phonology, the term ‘articulation’ refers to the configuration which produces the sounds of speech while both consonant and vowel belong to phoneme category.
A) As you pointed out, we use the word “consonant” instead of articulation or articulation methods.
2. Page 2, line 4 of the countdown. “contain meaning (eg, /ka/, /nu/, /si/)”, punctuation missing?
A) We corrected the missing punctuation.
3. 3. Page 2, line 3 of the countdown. “impact on However,”, typo?
A) We corrected the wrong upper case letter(However) to lower case letter(however).
4. Page 4, line 8-9 of the countdown. From a linguistic point of view, it is more appropriate to categorized them with vowel, consonant and tone). Articulation methods seem strange. You are suggested not to use this term as ‘consonant’ is more accurate, in accordance with vowel and other segmental or supra-segmental units. For the same consideration, ‘tone’ is proposed to replace to ‘frequencies’.
A) As you pointed out, we modified “consonant and tone” to replace to “articulation and frequencies”.
5. Page 15, line 11. “see. In fact,”, typo?
A) We deleted the typo error.
6. Page 15, line 5-7 of the countdown. What do you mean by “The syllable consonants in Korean are 7 of ㅂ/p/, ㄷ/t/, ㄱ/k/, ㅁ/m/, ㄴ/n/, ㅇ/ ŋ, ㄹ/l, while Chinese is only 2 of /-n/ and / ŋ/.”? Please check and confirm. I guess you are mentioning the coda types differences between Korean and Chinese?
A) We appreciate for pointing out the error. We modified the sentence to convey the exact content.

**Response to the Reviewer #2**

1. the articulation of consonants consists of the place of articulation and the method of articulation. This paper only considers the method of articulation, so the author should clarify why they don’t take the effect of the place of consonants into account.
A) Because of the classification of consonants varies depending on the language, we considered only plain consonant, aspirated consonant, and voiced consonant which is a common consonant in many languages and known to give a similar emotional impression in this study. The concepts that were not fully considered in this study were mentioned as limitations of the study.
2. this paper simply using nationality as the factor of cultural differences, however, the Chinese participants in this paper are all living in Korea, the length of time living in Korea may affect the result.
A) This study was conducted on Chinese people who came to live in Korea after entering a Korean university. They are Chinese who have been introduced to Korean culture since they became adults, it is assumed that they have a unique cultural characteristic acquired in the process of growing up even if they have become familiar with Korean culture. However, as you pointed out, all the levels of Korean cultural experience of each participant had not controlled. But this study is the first study to compare the difference in perception between arousal and valence values for Korean language, so we discussed it in terms of limitations so that it can be fully considered in future studies.
3. Chinese has at least seven dialects including Cantonese, Min, Hakka, etc. The southern dialects are quite different from Mandarin. In this paper, the authors do not consider the different dialectal backgrounds of the participants or at least not clarify this point. For example, most southern Chinese dialects have -m, -n, -ŋ, -p, -t, -k coda. So, in the discussion section, the discussions on the differences of Korean and Chinese is only true for Mandarin Chinese.

A) In this study, only Chinese who use Mandarin were required to response to the questionnaire when distributing the questionnaire. This was not specified in the paper, so the relevant content was written reflecting what you pointed out.

4. since the experiment was conducted online, the outside environments are not the same for all participants. This will somehow affect the result since the researchers can’t clearly see how the participants finish the experiments. Are there any data of participants are invalid and how the distinguish? The experiment done in this paper has shed some lights on the difference of emotions felt toward Hangul phonemes between Korean and Chinese women. The authors should control the experiment environments, and consider more about the dialectal backgrounds of participants, so as to draw a rigorous conclusion.
A) Although the experiment was conducted online, the environment of all participants was not controlled equally, but we emphasized the notice of the main points before participating in the experiment as follows: 1) Set the time to run the experiment for 1 hour (do not interrupt this experiment in the middle), 2) the surrounding environment is set so that it is not affected by external stimuli (e.g., noise), 3) recommend using earphones and check the computer volume of device in advance.

Since this study was intended to measure the emotional response to auditory stimuli presented, it was assumed that the participants’ basic emotional state would have a more significant influence, although the surrounding environmental factors were important at the time of the experiment. Accordingly, the emotional state of each participant was measured in advance, and as a result, it was confirmed that there was no difference in the emotional state of the participants.

5. Typo errors: p10: “Cultural differences can affect the perception of morphemes with meaning (eg, happiness, anger, fear), but the smallest sound unit that does not contain meaning (eg, /ka/, /nu/, /si/) It is easy to expect that cultural differences will not have an impact on However”
A) We corrected as “Cultural differences can affect the perception of morphemes with meaning (eg, happiness, anger, fear), but the smallest sound unit that does not contain meaning (eg, /ka/, /nu/, /si/). It is easy to expect that cultural differences will not have an impact on however,”
