# Supplementary material for: Differences in arousal and valence on the Korean phoneme of artificial voice between Korean and Chinese women
Source: PLoS One. 2023 Apr 7;18(4):e0284045. doi: 10.1371/journal.pone.0284045 (PMC10081760; doi:10.1371/journal.pone.0284045)
Supplement: Attachment — Submitted filename: Response to reviewers.docx [file pone.0284045.s003.docx]

**Review Comments to the Author (Reviewer #2)**

We appreciate your sincere comments.

1. There is no voiced consonant in Mandarin Chinese, the result find in the paper shows that ‘line 216-218 Korean reported higher arousal than Chinese in lenis and aspirated consonant, whereas there was no distinct difference between nationalities in voiced consonant’. So, the phonetic system (inventories) of Mandarin may affect the reaction of Chinese women in this experiment, since lenis and voiced consonants are only phonetic variants, but not phoneme in Mandarin. I think this point is more pivotal than the differences between end sounds in Korean and Chinese as the author mention. But in the discussion part, the authors do not mention the lack of voiced consonant in Mandarin.
A) We added discussion as followed: Koreans showed higher arousal than Chinese in lenis and aspirated consonants, while there was no clear difference between nationalities in voiced consonants. In general, Mandarin Chinese does not use voiced and voiceless sounds as discriminator as in Korean, so how well Chinese and Korean distinguish between voiced and aspirated sounds does not seem to affect the valence of audible sounds. However, the response values to the voiced consonants collected in this study tended to be somewhat lower in both Koreans and Chinese compared to lenis and aspirated consonants. In other words, it is estimated that the sound accompanying the vocal cords like voiced consonants lowered the arousal level, while it played a role in activating valence level.
2. Although the authors mention three limitations at last, we should be aware that all these limitations make the present paper less convincing. I suggest the authors to conduct the same experiment on male participants and see whether the result is the same with the women’.
A) We appreciate for your sincere opinion. In this study, only one gender was selected that could be considered similar in response level or pattern, assuming that there may be a difference in response according to experience depending on the emotional state. Therefore, we tried to match the homogeneity of the emotional state or level within the group as much as possible. However, as we also pointed out in our paper, we will conduct further studies on men with the same experiment, since it is assumed that gender differences may exist according to cultures. We are currently preparing for a specific experiment and will implement it soon.
3. Below is one confusing sentence and the authors need to double check. (1) p3 line 58, For example, it is assumed that the word for dog in thousands of languages will use consonants and vowels to symbolize large entities, and consonants and vowels to symbolize relatively small entities for ants.
A) We modified the sentence as: For example, in thousands of languages, the word ‘dog’ tends to use consonants and vowels with sounds that can represent relatively large entities, while consonants and vowels with smaller sounds can be used to symbolize relatively small entities, such as ‘ant’.
4. Some typos: (1) p4 line 80, According to the existing claim that all sounds have acoustic characteristics such as ‘rough or soft’, strong or weak’… [only one ’ ] (2) p8 line 157, measures ''trait anxiety'', it is better to use ‘’ in English instead of “”.
A) We corrected all the typos you found.
